# IgA binds to the AD‐2 epitope of glycoprotein B and neutralizes human cytomegalovirus

**DOI:** 10.1111/imm.13286

**Published:** 2020-12-13

**Authors:** Saima Siddiqui, Sarah Hackl, Hamid Ghoddusi, Megan R. McIntosh, Ariane C. Gomes, Joshua Ho, Matthew B. Reeves, Gary R. McLean

**Affiliations:** ^1^ Cellular and Molecular Immunology Research Centre London Metropolitan University London UK; ^2^ Microbiology Research Unit London Metropolitan University London UK; ^3^ Institute for Immunity and Transplantation University College London London UK; ^4^ National Heart and Lung Institute Imperial College London London UK

**Keywords:** AD‐2 epitope, glycoprotein B, human cytomegalovirus, immunoglobulin A

## Abstract

Human cytomegalovirus (HCMV) is a ubiquitous pathogen that is potentially pathogenic in immunosuppressed individuals and pregnant females during primary infection. The HCMV envelope glycoprotein B (gB) facilitates viral entry into all cell types and induces a potent immune response. AD‐2 epitope is a highly conserved linear neutralizing epitope of gB and a critical target for antibodies; however, only 50% of sero‐positive individuals make IgG antibodies to this site and IgA responses have not been fully investigated. This study aimed to compare IgG and IgA responses against gB and the AD‐2 epitope in naturally exposed individuals and those receiving a recombinant gB/MF59 adjuvant vaccine. Thus, vaccination of sero‐positive individuals improved pre‐existing gB‐specific IgA and IgG levels and induced de novo gB‐specific IgA and IgG responses in sero‐negative recipients. Pre‐existing AD‐2 IgG and IgA responses were boosted with vaccination, but de novo AD‐2 responses were not detected. Naturally exposed individuals had dominant IgG responses towards gB and AD‐2 compared with weaker and variable IgA responses, although a significant IgA binding response to AD‐2 was observed within human breastmilk samples. All antibodies binding AD‐2 contained kappa light chains, whereas balanced kappa/lambda light chain usage was found for those binding to gB. V region‐matched AD‐2‐specific recombinant IgG and IgA bound both to gB and to AD‐2 and neutralized HCMV infection in vitro. Overall, these results indicate that although human IgG responses dominate, IgA class antibodies against AD‐2 are a significant component of human milk, which may function to protect neonates from HCMV.

## Graphical abstract

Human IgG and IgA responses to HCMV gB and AD‐2 epitope were determined. IgG to gB dominates, and a reduced frequency of AD‐2 responses, including IgA, is observed in serum and saliva, but IgA to AD‐2 dominates in human breastmilk. Vaccination studies demonstrated that IgA to gB could be induced but that IgA to AD‐2 was only induced in sero‐positive individuals.

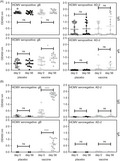

**Abbreviations**
Ab: antibody
AD: antigenic determinant
ARPE‐19: human retinal pigment epithelial cells
C: constant region
DMEM: Dulbecco's modified Eagle's minimal essential medium
ELISA: enzyme‐linked immunosorbent assay
FACS: fluorescence‐activated cell sorting
FCS: fetal calf serum
FITC: fluorescein isothiocyanate
gB: glycoprotein B
HCMV: human cytomegalovirus
HEK 293: human embryonic kidney cells
HFF: human foreskin fibroblasts
IE: immediate early
IgA: immunoglobulin A
IgG: immunoglobulin G
mAbs: monoclonal antibodies
MOI: multiplicity of infection
OD: optical density
PBS: phosphate‐buffered saline
pHC: immunoglobulin heavy chain plasmid
pLC: immunoglobulin light chain plasmid
sIgA: secretory IgA
Vh: heavy chain variable region
Vk: light chain variable region

## INTRODUCTION

Human cytomegalovirus (HCMV) is a ubiquitous pathogen that affects approximately 85% of the global human population.^1^ HCMV infection is resolved naturally in healthy individuals due to a rigorous immune response, but this opportunistic pathogen remains a substantial cause of morbidity and occasional mortality in immunocompromised patients such as transplant recipients,^2^ in AIDS patients^3^ and in fetuses.^4^, ^5^ Consequently, this clinical burden has led to the application of antiviral therapies,^6^ antibody‐based treatments^7^ and a high priority placed on vaccine development for HCMV intervention.^8^ Limitations of these therapies and prophylactics have necessitated a deeper understanding of the HCMV life cycle, viral structural determinants and host immune correlates of protection. This in turn has led to the development in recent years of numerous monoclonal antibodies (mAbs) and novel vaccine candidates for HCMV.

Although hyperimmune globulin preparations have shown mixed success in the prevention of congenital HCMV infection^9^ and HCMV disease in solid organ transplantation,^10^ the increasing use of mAbs as therapeutics and prophylactics for human diseases^11^ has spurred interest in the development of antiviral mAbs, including for HCMV.^7^ Numerous mAbs are under preclinical development and target diverse structural components of the virus that are required for entry into cells. The major viral targets of mAbs and host humoral immunity are the glycoprotein B (gB) and the pentamer complex consisting of gH, gL, pUL128, pUL130 and pUL131A. Abs to these viral components are known to block infection of fibroblasts, epithelial cells, endothelial cells, macrophages and dendritic cells.^12^, ^13^ Most mAbs studied are of the IgG isotype despite increasing evidence of the importance of IgA, particularly at mucosal surfaces.^14^ Humans have two subclasses of IgA, with serum consisting of 90% of IgA1 and 10% IgA2, whereas in mucosal samples such as saliva and breastmilk, the ratio is closer to 40:60.^14^ Furthermore, multiple molecular forms of IgA exist. In human serum, IgA is predominantly monomeric, whilst the mucosal secretory IgA (sIgA) is dimeric. It is predicted that IgA mAbs will be used more frequently in the future and rival the therapeutic and prophylactic uses of IgG.

HCMV gB is a highly conserved glycoprotein among the herpesvirus family and plays a critical role in infectivity and cell‐to‐cell spread being the viral determinant critical for membrane fusion during entry.^15^ Based on this critical role of gB in facilitating virus entry and being a well‐described immune system target, gB is a promising candidate for development of vaccines and Ab‐mediated therapies. Five antigenic determinants or epitopes (termed AD‐1, AD‐2, AD‐3, AD‐4 and AD‐5) located on gB have been described.^16^ The conformational AD‐1 epitope was originally described as the major epitope,^17^ inducing production of IgG Abs in all infected individuals; however, not all of the AD‐1 epitope binding Abs are neutralizing.^18^ Conversely, only 50% of sero‐positive individuals are capable of generating AD‐2‐specific Abs in spite of their potent neutralizing capability^19^ and all human mAbs identified to this epitope are of the IgG subclass and have been shown to consist of kappa light chains.^16^, ^20^, ^21^ The use of kappa light chains is thought to restrict binding properties of human Abs to simpler epitope structures such as AD‐2.^22^ More recently, the AD‐4 and AD‐5 epitopes, which are conserved and immunogenic and induce potent neutralizing Abs, have been identified on gB.^16^ The AD‐3 epitope was identified within the gB cytoplasmic domain and is a target of non‐neutralizing Abs.^16^ Thus, the knowledge of gB epitope‐based Abs has increased in recent years and coupled with the development of recombinant gB as a subunit vaccine^23^ demonstrates its importance.

At present, there is no licensed vaccine for HCMV; however, the recombinant gB subunit vaccine has been evaluated extensively in recent years through numerous clinical trials. Studies have demonstrated immunogenicity and safety, including the boosting of pre‐existing immunity in those already infected with HCMV.^24^, ^25^, ^26^, ^27^, ^28^ Recombinant gB vaccination has displayed partial efficacy through reductions in primary maternal infection, which would likely reduce the incidence of congenital infections,^29^ and was effective at reducing viraemia when administered to kidney and liver transplant patients – particularly in sero‐negative vaccine recipients.^30^ Despite these encouraging clinical trials where 50% vaccine efficacy was observed, the mechanistic correlate of protection for the gB vaccine remains elusive. Studies of sera taken from HCMV‐sero‐negative recipients of the gB vaccine demonstrated limited evidence of neutralizing antibodies^31^, ^32^ although in the transplant study, challenge of vaccinees with an organ from a HCMV‐sero‐positive donor did reveal a rapid accumulation of gB‐specific neutralizing antibodies post‐transplant in some patients. In contrast, individuals naturally infected with HCMV do generate neutralizing antibody responses directed against epitopes within gB including AD‐2. Interestingly, two separate studies have suggested that AD‐2 responses in naturally infected individuals are an important correlate of protection,^33^, ^34^ and vaccination with gB will boost this response in the 50% of individuals who have generated it in response to prior natural infection.^35^

Much of these prior studies have focused on the IgG response to gB and thus not fully assessing the entire repertoire of antibody responses to HCMV and vaccination. In this study, we have investigated the contribution of IgA alongside IgG Abs to gB and AD‐2 generated by vaccination with recombinant gB and in healthy adults naturally exposed to HCMV. We report data obtained for serum IgG and IgA responses to gB and AD‐2 in gB/MF59 adjuvant vaccine recipients, matched human serum and saliva samples from individuals naturally exposed to HCMV, human breastmilk samples and recombinant Abs specific for AD‐2 formatted as human IgA or IgG. Our data indicate that whilst human IgG responses to gB and AD‐2 predominate, substantial IgA responses were detected, particularly in breastmilk. The demonstration that recombinant monoclonal IgA antibodies directed against AD‐2 can neutralize HCMV infection in vitro argues that IgA antibodies should be considered another component of protective immunity to HCMV via AD‐2.

## MATERIALS AND METHODS

### Antigens

The following HCMV‐specific antigens were used: gB recombinant protein, stock concentration 0.8 mg/ml, was also used as the vaccine immunogen, provided by Sanofi Pasteur; AD‐2 is a short linear peptide made by solid‐phase synthesis, stock concentration 1 mg/ml; and sequence SHRAN**ETIYNTTLKY**GDKL (minimal epitope shown bold) was synthesized and purchased from Alta Biosciences, UK.

### Human sample collection, processing and storage

Human serum samples were obtained from a prior vaccine trial of recombinant gB from HCMV and MF59 adjuvant trial, performed on a group of solid organ transplant patients (NCT00299260) enrolled in a phase 2 randomized and double‐blinded placebo‐controlled study.^30^ Samples were obtained from placebo or vaccinated volunteers at day 0 (baseline and first dose) and day 56 (1 month afters econd dose), were blinded and were stored at −80°C until use.

Twenty‐four healthy adult volunteer participants each donated both blood and saliva samples for matching purposes. Blood samples (5 ml) were collected in sterile tubes (without anticoagulant) and then left to clot. The samples were centrifuged at room temperature at 280 *g* for 15 min, and the serum fraction was separated from the clot. Serum was stored at −80°C prior to analysis. Volunteers also donated 2 ml of saliva, which was centrifuged at 5040 *g* for 10 min at 4°C to remove cells and debris. The supernatant was collected and stored at −80°C prior to analysis. Participants were anonymized by coding the samples provided, thereby maintaining patient confidentiality, safety and respect. Exclusion criteria included the following: volunteers suffering from any acute or chronic disease; age <18 years; and pregnancy. The study was approved by the Research Ethics Committee of the School of Human Sciences of London Metropolitan University.

Fourteen human breastmilk samples, originally collected from milk bank at Queen Charlotte's and Chelsea Hospital, Imperial College Healthcare NHS Trust, were kindly provided by the Microbiology Research Unit of London Metropolitan University. Each sample was vortexed briefly to dissolve fats and divided into four aliquots and stored at −80°C before analysis.

### ELISA

ELISA was performed as described earlier^20^, ^33^ with the modification that coating with antigens to determine specific binding (AD‐2 peptides or recombinant gB) or capture antibodies to determine levels of IgG and IgA (anti‐human kappa and anti‐human lambda) diluted in PBS was performed overnight at 4°C. Antigens and capture antibodies were coated at a final concentration of 1 µg/ml. In all cases, blocking was performed with 5% non‐fat dry milk in PBS containing 0.1% Tween‐20 (PBST milk) at 37°C for 2 h. Samples diluted in PBST milk were added, and plates were incubated overnight at 4°C. Peroxidase‐labelled secondary Abs to human IgG, human IgA, human kappa and human lambda chains (Southern Biotechnology Associates) were diluted in PBST milk and added to plates, which were incubated at 37°C for 2 h. Colour was developed following the addition of 3,3′,5,5′‐tetramethylbenzidine (TMB; Thermo Fisher) and stopped with 0.1 M HCl. Washing was performed between steps using Skatron SkanWasher 300, and the optical density at 450 nm was determined using an OMEGA plate reader (BMG Labtech).

### Human recombinant monoclonal antibodies

8F9, QG1 and FA9 are three human monoclonal IgG, which bind to the AD‐2 epitope of gB.^20^ The L and H chain V region cDNA of each was inserted into the human Ig expression vectors, pLC‐huCκ, pHC‐huCα1 and pHC‐huCγ1 expression vectors,^36^ for expression as human IgA1 and human IgG1 mAbs. L chain and H chain plasmids containing 8F9, QG1 and FA9 cDNA were propagated in *E*. *coli* after transformation and selection from LB–ampicillin plates, where single colonies were chosen and grown overnight at 37°C. Plasmids were isolated from bacterial cultures using Qiagen plasmid kits. Restriction digest was performed to confirm the plasmid integrity followed by Sanger sequencing to confirm correct Ab V regions. Plasmid concentrations were determined by NanoDrop UV spectrophotometry.

### Transient transfection of HEK 293 cells

HEK 293 cells were seeded in a 6‐well plate at 6.25 × 10^5^ cells per well in 2 ml of Dulbecco's modified Eagle's minimal essential medium (DMEM; Invitrogen) supplemented with 2% penicillin–streptomycin (Invitrogen) and 10% FCS (Invitrogen) the day before transfection. On the day of transfection, media were replaced with 0.5 ml of Opti‐MEM. For each well of cells, 5 µl of Lipofectamine 3000 reagent was diluted into 125 µl of Opti‐MEM™ and gently mixed. 5 µg of plasmid DNA (equal amounts of pLC and pHC) was diluted in Opti‐MEM™ including 5 µl of P3000 reagent and mixed well. Diluted Lipofectamine 3000 reagent was combined with each tube of diluted DNA (1:1 ratio) and incubated at room temperature for 10–15 min to form DNA–Lipofectamine 3000 reagent complexes. After incubation, the complexes were added to each well containing cells and gently mixed by rocking the plate back and forth. After 4–5 h of incubation, DMEM‐FCS was added and cells were incubated at 37°C at 5% CO_2_ concentration for 4–5 days with media harvested and added each day. Supernatants were verified for monoclonal antibody expression by ELISA prior to further experiments.

### HCMV neutralization assays

Human foreskin fibroblasts (HFFs) were seeded in 96‐well cell culture plate, and a confluent monolayer was allowed to form. Virus stocks (TCID50 of 5.44 × 10^6^/ml of high passage Merlin strain) were diluted and added to infect the cells at a MOI of 1. Next, 100 µl of each mAb (10 µg/ml) was mixed with virus in triplicate, titrated and incubated for 30 min at 37°C. The antibody–virus mixture was then added to HFFs and incubated for 1 h at 37°C. The inoculum was removed, and growth medium was added and incubated overnight at 37°C before the cells were fixed with cold, 100% ethanol (−20°C) for 30 min, washed three times with PBS and stained for IE gene expression using anti‐IE (Millipore; 1:1000) for 1 h at room temperature. Unbound antibody was removed by washing three times with PBS, and reaction wells were stained with goat anti‐mouse Alexa Fluor 568 nm (Life Technologies; 1:2000) for 1 h at room temperature. Nuclei were counterstained with 4′,6‐diamidino‐2‐phenylindole (DAPI). Unbound antibody was removed by washing three times with PBS, and percentage infection was enumerated by immunofluorescence imaging using Hermes WiScan instruments. A similar method was employed using human retinal pigment epithelial cells (ARPE‐19) as the target of infection. Here, we pre‐mixed IgA1 mAbs (diluted 1:3) with the epithelial–tropic strain of HCMV (TB40/e) before following the procedure described above for HFFs.

### Flow cytometry analysis

Mouse myeloma NS0 cells and engineered NS0 cells stably expressing the N‐terminus of gB (residues 28‐100) containing the AD‐2 epitope on their surface as described^20^ were suspended at ~10^6^/ml in PBS containing 0.5% FCS (FACS buffer) and then incubated with Abs for 30 min on ice. Following incubation, the cells were washed with ice‐cold FACS buffer and then stained with diluted anti‐human к chain FITC conjugate (Southern Biotechnology Associates) for 30 min on ice in dark. Fluorescence was analysed using a Guava 8HT cytometer, and the data were analysed using FCS Express (De Novo software).

### Statistical analyses

The analysis of data obtained was performed using GraphPad Prism software. Data were first evaluated for normal distribution to ascertain which statistical tests should be applied. Statistical differences between the mean OD values of antibodies at each dilution were obtained from the Mann–Whitney test (ns, not significant; *P < 0.05; **P < 0.005; ***P < 0.001; and ****P < 0.0001). Correlations were assessed by Spearman's correlation and the *r* values and *P* values stated.

## RESULTS

### IgG and IgA responses to HCMV gB and AD‐2 in HCMV‐sero‐positive volunteers

**Figure 1:**
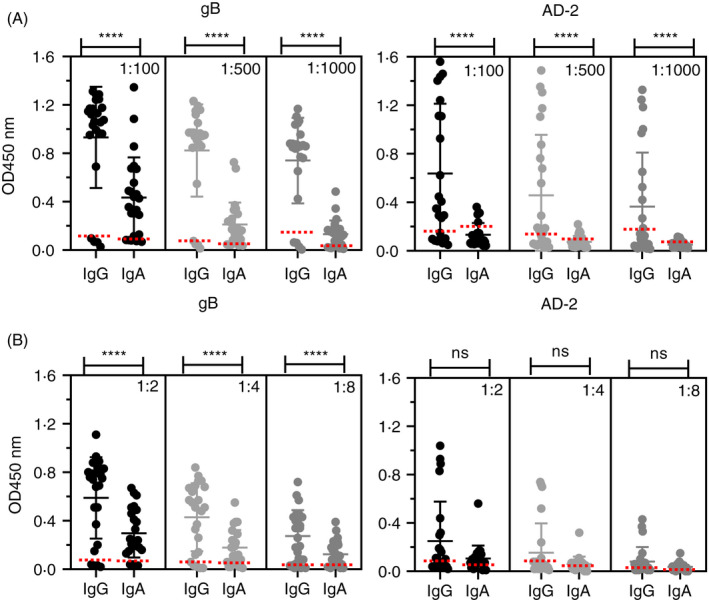
Human IgG and IgA bind HCMV gB and AD‐2. ELISA to determine IgG and IgA binding to gB and AD‐2 epitope of HCMV in (A) human serum (*n* = 24) and (B) human saliva (*n* = 24). All samples were diluted as indicated, and data are represented as OD450 nm values of triplicate determinations of three independent experiments. Horizontal dotted lines representing positive/negative cut‐off values for negative gB and negative AD‐2 samples were based on IgG gB‐negative samples (*n* = 4). Statistical differences comparing the mean OD450 nm values of IgG and IgA binding to gB and AD‐2 epitope at each dilution were obtained from the Mann–Whitney test (ns, not significant; ****P < 0.0001)

Serum and saliva samples were obtained from 24 healthy adult volunteers and screened for binding levels of IgG and IgA to recombinant gB and the AD‐2 epitope (Figure 1). Four samples were found to be negative as defined by lack of serum IgG to gB when diluted 1:100, and these samples were used to define the positive–negative cut‐off values for each Ab isotype binding to gB and AD‐2 (Figure S1). For serum samples, IgG responses to gB were the strongest with 20 positive samples (83.3% positive), whereas the IgA responses were much more variable and titrated out earlier than IgG although 19 samples (79.16%) remained positive at 1:100 dilution (Figure 1A). Serum IgG and IgA responses to AD‐2 were weaker than those to gB, with 16 samples IgG positive (66.66%), but only four samples were determined as IgA positive at 1:100 dilution (20.8%) (Figure 1A). In general, the saliva results (Figure 1B) display similar trends as those observed with serum (Figure 1A). For gB, binding of IgA was significantly weaker than IgG, and for AD‐2, a reduced frequency of positive samples was noted.

**Figure 2:**
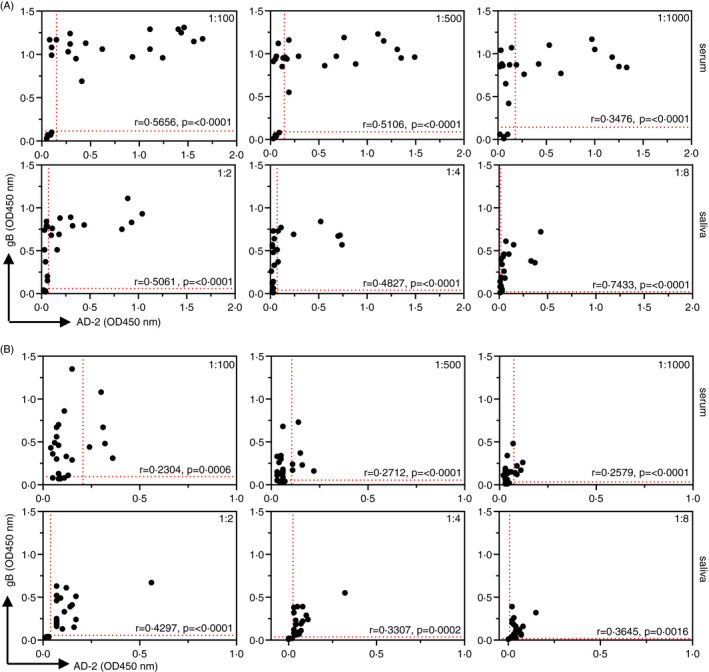
Scatterplot correlations of individual samples displaying IgG and IgA binding to both HCMV gB and the AD‐2 epitope. ELISA data displaying comparative IgG (A) and IgA (B) binding to gB and AD‐2 epitope of HCMV in each sample of human serum (*n* = 24) and human saliva (*n* = 24). Samples were diluted as indicated, and data are represented as OD450 nm values of triplicate determinations of three independent experiments. Horizontal and vertical dotted lines representing positive/negative cut‐off values for negative gB and negative AD‐2 samples, respectively, were based on IgG gB‐negative samples (*n* = 4). Correlation coefficients (Spearman's) and statistical significance for each set of determinations comparing serum or saliva IgG and IgA binding to gB and AD‐2 epitope are displayed within each graph

Representation of these data as scatterplot correlations allowed us to compare individual IgG and IgA responses against gB and AD‐2 together (Figure 2). The strongest correlations were observed with serum and saliva IgG responses with maximal *r* values of 0.5656 and 0.7433, respectively (Figure 2A). Correlations were lower for both serum and saliva IgA responses (Figure 2B). These data most likely reflect the differences in relative levels of each Ab isotype between saliva and serum, with serum containing excess IgG over IgA and saliva more balanced levels.^37^ These data are also consistent with AD‐2 responses being extremely variable between individuals, whereas gB responses are reliably stronger, reflecting the number of possibilities that polyclonal Abs have to bind the much larger gB molecule with its additional available epitopes. Thus, only a proportion of individuals generate IgG and IgA to the AD‐2 epitope despite being considered HCMV‐sero‐positive.

### IgG and IgA responses to HCMV gB and AD‐2 in recombinant gB vaccine recipients

**Figure 3:**
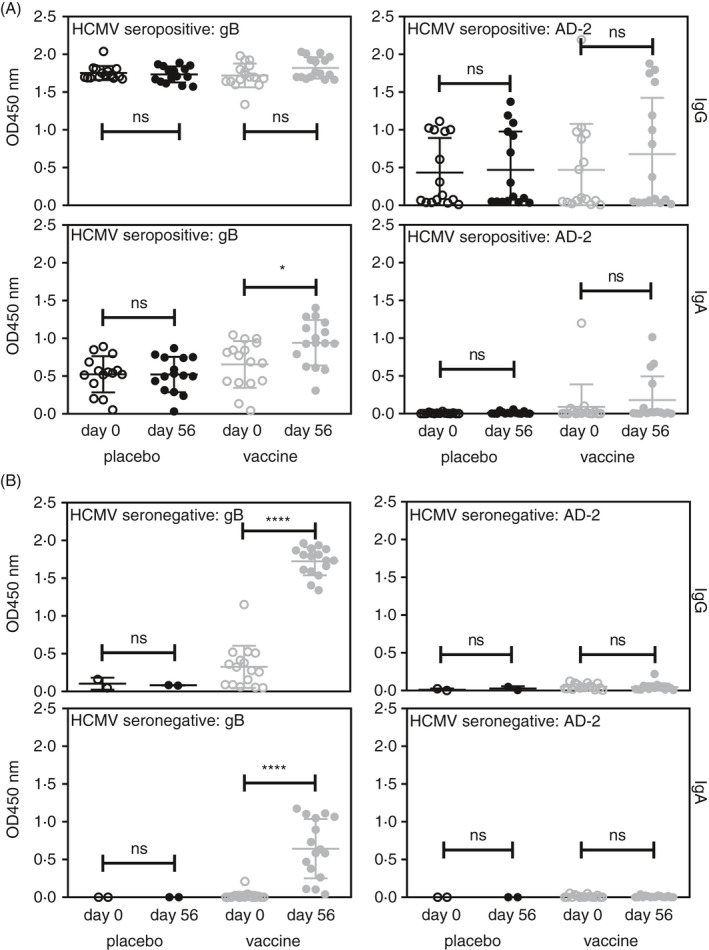
gB vaccination induces IgG and IgA responses to gB and AD‐2 in sero‐positive recipients. Serum samples obtained from the recombinant gB vaccination trial were analysed for IgG and IgA binding to gB and AD‐2 by ELISA. Individuals were stratified into (A) sero‐positive (IgG+to gB) and (B) sero‐negative (IgG‐ to gB). Statistical differences between the mean OD values of day 0 and 56 of vaccine recipients for IgG and IgA for binding to gB and AD‐2 epitope were obtained from the Mann–Whitney test (ns, not significant; *P < 0.05; ****P < 0.0001). Placebo groups showed no significant differences

To compare natural infection with vaccination, we investigated human serum samples obtained from a prior vaccine trial of recombinant gB from HCMV^30^ for IgG and IgA responses to HCMV gB and the AD‐2 epitope. Findings for IgG responses had been previously published^33^ and demonstrated that levels of IgG to AD‐2 correlated with a reduced incidence of viraemia in HCMV‐sero‐positive transplant recipients. Importantly, whilst the gB vaccine could boost pre‐existing IgG responses against AD‐2, de novo IgG responses were not detected^27^. We therefore decided to investigate whether these data with IgG were reflected with the IgA isotype response. Figure 3 displays our findings investigating serum IgG and IgA responses to gB and the AD‐2 epitope for placebo and vaccine recipients, stratified by HCMV‐sero‐positive status. The gB/MF59 vaccine boosted gB binding Abs in sero‐positive recipients only in the IgA responses, which reached statistical significance (Figure 3A). IgG and IgA responses to AD‐2 showed a trend towards boosting (Figure 3A) but did not reach statistical significance. However, if individuals were stratified into AD‐2 responders (based on one sample being considered positive according to cut‐off values) and removed the AD‐2 non‐responders (no positive AD‐2 sample), the vaccine boosted pre‐existing AD‐2 responses (Figure S2). Thus, AD‐2 responses do not develop in all subjects and gB vaccination can boost these responses only in sero‐positive subjects who have already developed AD‐2 responses after natural infection. In sero‐negative gB vaccine recipients, robust IgG and variable IgA responses to gB were also detected. However, consistent with previous studies, IgG responses to the linear AD‐2 epitope were not induced by vaccine and similar results were obtained with IgA (Figure 3B). Therefore, vaccination with gB does not elicit de novo AD‐2 responses in sero‐negative or sero‐positive subjects. In conclusion, these new findings assessing IgA responses in response to gB/MF59 match those previously found for IgG.^33^ Finally, analysis of individuals' responses revealed that all the IgA‐positive samples to AD‐2 were also IgG‐positive.

### Human milk IgG and IgA display different binding profiles to gB and AD‐2

**Figure 4:**
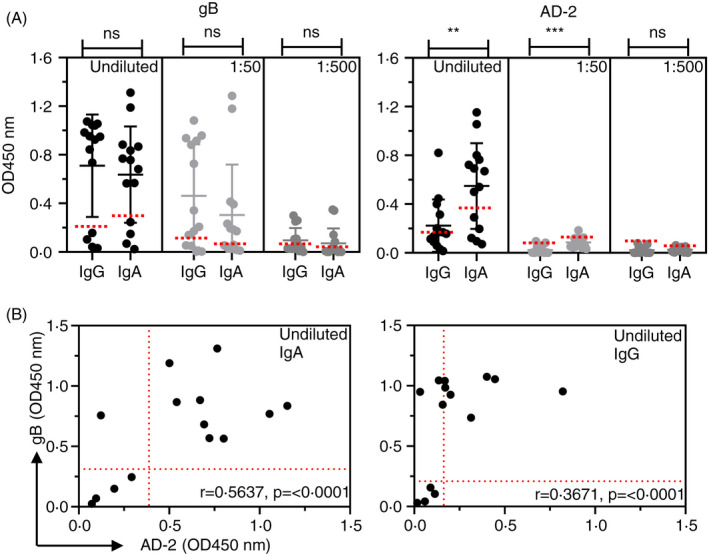
Human milk‐derived IgG and IgA bind HCMV gB and AD‐2. (A) ELISA to determine IgG and IgA binding to gB and AD‐2 epitope of HCMV in human breastmilk samples (*n* = 14). Samples were diluted as indicated, and data are represented as OD450 nm values of triplicate determinations of three independent experiments. Horizontal dotted lines representing positive/negative cut‐off values for negative gB and negative AD‐2 samples were based on IgG gB‐negative samples (*n* = 4). Statistical differences comparing the mean OD450 nm values of IgG and IgA binding to gB and AD‐2 epitope at each dilution in part A were obtained from the Mann–Whitney test (ns, not significant; **P < 0.01; ***P < 0.001). (B) Scatterplot correlations of individual undiluted samples displaying IgG and IgA binding to both HCMV gB and the AD‐2 epitope. Horizontal and vertical dotted lines are shown representing positive/negative cut‐off values for negative gB and negative AD‐2 samples, respectively, and were based on IgG gB‐negative samples (*n* = 4). Correlation coefficients (Spearman's) and statistical significance for each set of determinations comparing IgG or IgA binding to gB and AD‐2 epitope are displayed within each graph

Thus far, we could detect IgA responses against both gB and AD‐2 in natural infection and that vaccination promoted IgA responses against gB. The studies in both sera and saliva also suggested that the IgG response predominated over IgA, so we next turned our attention to human antibody responses to HCMV found in breastmilk samples. Similar to human serum and saliva samples, we determined binding responses to gB and AD‐2 with data presented as dot plots and correlations in Figure 4. Fourteen samples were investigated. Again, we used four sero‐negative breastmilk samples to determine positive negative cut‐off values for the ELISA (Figure S3). For the remaining 10 samples, we observed similar binding profiles of IgG and IgA to gB that titrated out at 1:500 (Figure 4A). Interestingly, for AD‐2 binding, we found that IgA responses were significantly stronger than IgG (Figure 4A) in contrast to those observed with serum and saliva. The scatterplot correlations comparing individual samples binding response to gB and AD‐2 confirmed these data and demonstrated that the best (*r* = 0.5637) is seen with IgA responses (Figure 4B). Thus, the IgG/IgA picture in breastmilk was different to that seen in saliva and sera.

### Light chain usage by serum antibodies binding gB and AD‐2

**Figure 5:**
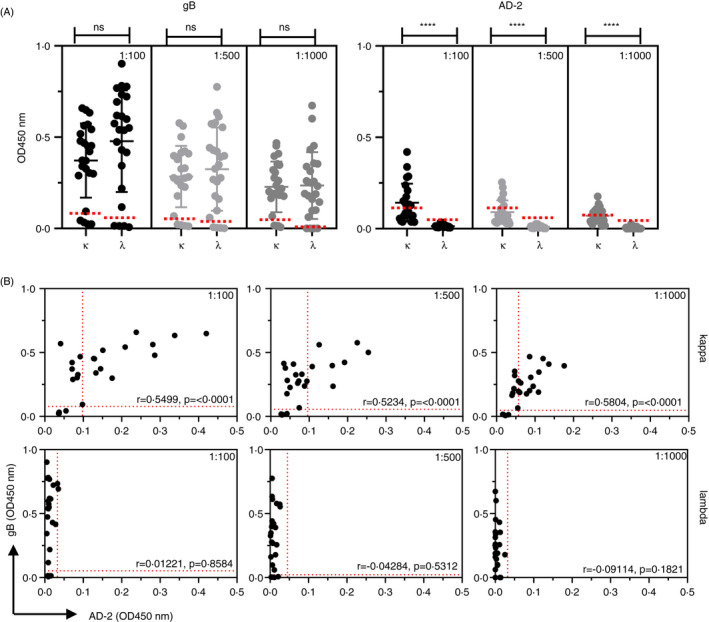
Human antibodies binding HCMV AD‐2 epitope preferentially use kappa (κ) light chains. (A) ELISA to determine light chain usage of Ig's binding to gB and AD‐2 epitope of HCMV human serum (*n* = 24). Horizontal dotted lines representing positive/negative cut‐off values for negative gB and negative AD‐2 samples were based on the IgG gB‐negative samples (*n* = 4). Statistical differences comparing the mean OD450 nm values for kappa (κ) and lambda (λ) chain use at each dilution were obtained from the Mann–Whitney test (ns, not significant; ****P < 0.0001). (B) ELISA data displaying comparative κ and λ usage in Ig's binding to gB and AD‐2 epitope of HCMV in serum samples. Horizontal and vertical and dotted lines representing positive/negative cut‐off values for negative gB and negative AD‐2 samples, respectively, were based on the IgG gB‐negative samples (*n* = 4). Correlation coefficients (Spearman's) and statistical significance for each set of determinations comparing κ or λ usage in Ig's binding to gB and AD‐2 epitope are displayed above each graph

Following the characterization of isotype responses in multiple compartments, we next sought to investigate the biology of AD‐2 antibodies in more detail. First, we characterized the contribution of the light chains to these antibodies found in human serum. We found that antibodies binding to gB displayed a balanced usage of kappa (κ) and lambda (λ) light chains, whereas the antibodies binding to AD‐2 were restricted to κ chain usage (Figure 5A). These data are confirmed within the scatterplot correlations comparing individual responses to gB and AD‐2 where the use of κ light chains displays *r* values ranging from 0.5234 to 0.5804, whereas λ light chain usage does not display significant values (Figure 5B). Similar results were observed when testing human milk samples for light chain usage in antibodies binding to gB and AD‐2 (Figure S4), suggesting that both IgG and IgA binding to AD‐2 prefers the use of κ chains.

### AD‐2‐specific recombinant antibodies expressed as IgG1 and IgA1

**Figure 6:**
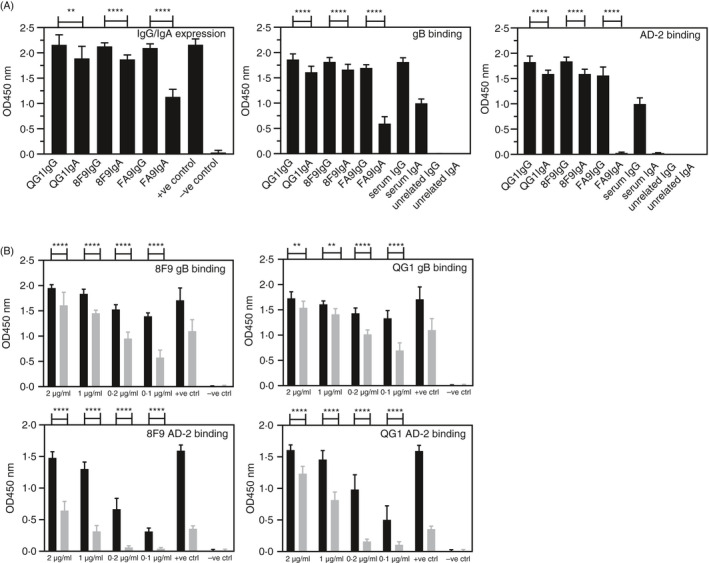
Recombinant antibodies expressed as human IgG and IgA bind gB and AD‐2 epitope of HCMV. (A) Expression of monoclonal recombinant antibodies QG1 IgG1, QG1 IgA1, 8F9 IgG1, 8F9 IgA1, FA9 IgG1 and FA9 IgA1 by transient transfection and ELISA of undiluted culture supernatants and binding to gB and AD‐2. +ve control, diluted human serum; –ve control, undiluted culture supernatant from untransfected cells; unrelated IgG and IgA are control human mAbs. (B) Binding and titration of concentration‐matched mAbs QG1 IgG1 (black bars), QG1 IgA1 (grey bars), 8F9 IgG1 (black bars), 8F9 IgA1 (grey bars) to gB and AD‐2 epitope of HCMV represented as OD values with human serum as positive control and unrelated IgG and unrelated IgA as negative control. Statistical differences between the mean OD values of IgG and IgA expression and for binding to gB and AD‐2 epitope were obtained from the Mann–Whitney test (**P < 0.01; ****P < 0.0001)

Using this information, we next expressed three human recombinant antibodies specific for AD‐2^20^ as both human IgG1 and IgA1 using immunoglobulin expression vectors (pHC‐huCγ1, pHC‐huCα1) and mammalian cell expression systems.^36^ Our data from Figure 5 and previous V region sequencing analyses^20^ demonstrated that all Abs binding AD‐2 used κ light chains, and therefore, we could construct intact V region‐matched human Abs by coexpression with pLC‐huCκ expression vectors. Figure 6 displays expression and binding data for recombinant immunoglobulins 8F9, QG1 and FA9. We first expressed these antibodies as both IgG1 and IgA1 and measured levels in culture supernatants and compared IgG1 and IgA1 binding against both gB and AD‐2. All were effectively expressed and bound to gB and AD‐2 as both IgG1 and IgA1 except for clone FA9 IgA1 (Figure 6A). We therefore decided to focus further studies on 8F9 and QG1 clones and normalize their concentrations as IgG1 and IgA1. These analyses revealed significantly stronger binding of IgG1 to both gB and AD‐2 as compared to IgA1 (Figure 6B). These data confirm that V region‐matched recombinant Abs show constant region‐dependent binding differences with IgG1 consistently stronger than IgA1, particularly when analysing binding to AD‐2.

### Recombinant IgG1 and IgA1 binding to cell‐associated gB and neutralization of HCMV in vitro

**Figure 7:**
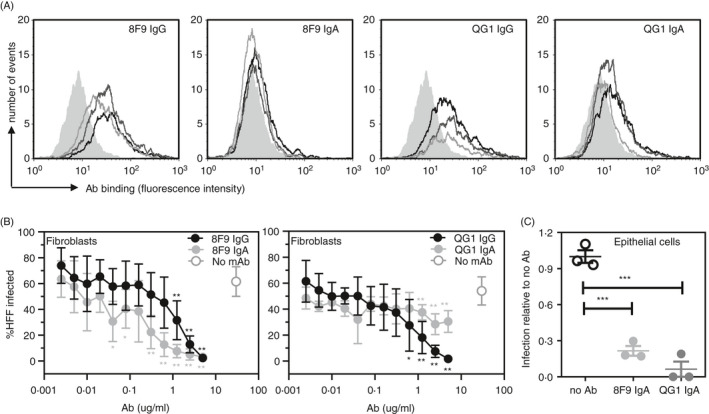
AD‐2 specific IgA mAbs neutralize HCMV in vitro. (A) Isotype‐matched mAbs 8F9 (IgG1 and IgA1) and QG1 (IgG1 and IgA1) binding to NS0 cells engineered to express the N‐terminal fragment of gB (NS0‐gBNT) as measured by flow cytometry. Grey filled histogram, secondary Ab alone: mAb – 2 µg/ml (black line), 1 µg/ml (dark grey line) and 0.2 µg/ml (light grey line). (B) Neutralization assays for HCMV infection of human foreskin fibroblasts (HFFs) represented as percentage of cells infected with high passage Merlin strain of HCMV. 8F9 IgG1, 8F9 IgA1, QG1 IgG1 and QG1 IgA1 were titrated with data shown as representative of 2 independently performed experiments. (C) Neutralization assay for HCMV (TB40/e) infection of human epithelial cells (ARPE‐19) represented as infection relative to no Ab control (assigned 1.0). 8F9 IgA1 and QG1 IgA1 were diluted 1:3 and assayed in triplicate. Statistical differences between the mean values of each mAb compared with no mAb for each concentration were obtained from the Mann–Whitney test (no asterisk is not significant; *P < 0.05; **P < 0.01; ***P < 0.001)

To confirm the data observed in ELISA binding experiments, we performed investigations using cells engineered to express an N‐terminal fragment of gB^20^ and compared AD‐2‐specific recombinant IgG and IgA binding. These experiments were designed to mimic what might be expected to occur for recognition via the AD‐2 epitope of cell‐associated gB. Figure 7A and Figure S5 display the flow cytometric analysis of recombinant IgG1 and IgA1 binding to cells expressing a fragment of gB. Binding of the 8F9 and QG1 clones expressed as IgG1 was observed, but very limited binding was detected when using the V region‐matched IgA1 variants. These data confirm that seen in ELISA experiments (Figure 6B) where reduced binding of the IgA1 clones to AD‐2 was noted. Despite the apparent reduced IgA1 binding to AD‐2, we found that the IgA1 was still able to neutralize HCMV infection in vitro (Figure 7B). In particular, 8F9 IgA1 neutralized at lower concentrations than 8F9 IgG1 (IC_50_ 0.2 vs. 1 µg/ml) and completely neutralized at 2 µg/ml. In contrast, QG1 IgA1 was less effective compared with its IgG1 counterpart (IC_50_ 3 vs. 0.8 µg/ml) and was unable to completely neutralize HCMV. Importantly, IgA1 antibodies 8F9 and QG1 also neutralized HCMV infection of epithelial cells (Figure 7C). These results suggest that the structural form of the AD‐2 epitope (within recombinant gB, synthetic peptide, cell‐surface expression, native virion) can influence recognition by Abs and, in particular, IgG versus IgA.

## DISCUSSION

In this study, through a comparison of human IgG and IgA responses to HCMV gB and AD‐2 epitope found in serum, saliva and breastmilk, we have investigated a potential role for IgA antibodies against HCMV infection. Despite relatively weak IgA responses and reduced frequency of binding to AD‐2 in matched human serum and saliva samples, we detected strong binding of IgA from breastmilk and recombinant IgA1 to AD‐2 bound effectively to both gB and AD‐2 and neutralized HCMV in vitro. Interestingly, we found that gB/MF59 vaccination boosted both IgG and IgA binding to gB and AD‐2 but only with pre‐existing responses for the latter. Our data are supportive of an important role for human IgA to gB and AD‐2 in the protective immune response against HCMV and warrant further investigation in gB vaccine trials.

Recombinant HCMV gB is under evaluation as a subunit vaccine to protect against congenital HCMV infection and transplant‐related viraemia. Serum Abs obtained from one such vaccine trial^30^ were used to probe the IgA responses to gB and AD‐2 epitope following gB/MF59 vaccination. Our results with IgA responses are consistent with those previously reported for IgG^33^ with vaccination inducing de novo IgA responses to gB in sero‐negative recipients and boosting IgA responses in sero‐positive recipients. In fact, gB vaccination reliably and significantly induced serum IgA responses to gB in both sero‐positive and sero‐negative recipients, suggesting that this component of the HCMV immune response is induced via the vaccine regimen. Prior analyses of epitope‐specific gB IgG responses in this transplant cohort revealed that AD‐2 Abs were linked with reduced viraemia and suggested that this might be a correlate of protection in HCMV‐sero‐positive solid organ transplant recipients.^33^ Importantly, vaccination with gB boosted these pre‐existing IgG AD‐2 responses but failed to induce de novo responses. We now show that this boosting effect also occurred in those individuals with pre‐existing IgA to AD‐2. The key question is whether IgA responses against AD2 are protective – however, given the number of patients with IgA AD‐2 responses, it was not possible to perform a retrospective clinical analysis of outcome. However, our in vitro neutralization data argue that IgA responses against AD‐2 could be protective. Thus, we propose that future studies of the humoral response to gB should include IgA analyses particularly in ongoing vaccine studies particularly those proposing to use AD‐2 as the immunogen.

Our data are consistent with previous studies that have shown that IgG Abs to AD‐2 in humans are known to be composed of a restricted set of Vh and Vκ regions,^20^, ^38^ which could be one reason why such Abs are rare and outnumbered by Abs binding other gB epitopes.^19^ This feature of AD‐2 Abs of both the IgG and IgA isotypes can also explain the low frequency of de novo synthesis of AD‐2 Abs following gB vaccination. Our current investigations have revealed new structural constraints of Abs binding to AD‐2. Here, we demonstrated that light chain usage in AD‐2‐specific Abs is restricted to use of κ light chains, whereas Abs binding to other epitopes of gB can consist of either κ or λ light chains. This feature of Abs binding short linear peptides is not unique to AD‐2 and has been demonstrated previously, with the preferential use of κ light chains a reflection of limited antigenic complexity.^22^ This could indicate another immune evasion mechanism of HCMV to restrict immune responses to an important conserved epitope required by the virus for cell entry.

To confirm our data with polyclonal Abs and to establish the significance of IgA to AD‐2, we created three V region‐matched AD‐2 specific mAbs as both IgG1 and IgA1. Our results with these mAbs demonstrated that even though IgG1 binding was stronger towards synthetic AD‐2 peptide, membrane‐associated AD‐2 epitope and recombinant gB, as compared to IgA1 binding, we found that IgA1 binding AD‐2 could efficiently neutralize HCMV in vitro. Thus, crucially, the IgA antibodies were clearly functional against HCMV. It is difficult to determine the contribution of IgA within polyclonal preparations where competition between IgG and IgA might be expected; therefore, our mAb studies are important to establish the relative importance of IgA responses to HCMV. Our data may reflect the natural binding preferences among the different Ab isotypes including C region effects on affinity and avidity for antigens observed previously.^39^ Most importantly, our data demonstrate that IgA is capable of binding to AD‐2, particularly in the absence of IgG, and strengthen our observations that IgA in human breastmilk displays superior binding over IgG.

It is important that human IgA binding AD‐2 was capable of neutralizing HCMV in vitro. In fact, the best neutralizing recombinant clone was 8F9 expressed as human IgA1. It displayed improved neutralizing capabilities over its IgG1 counterpart, requiring lower concentrations to achieve significant neutralization effects. Surprisingly, this particular Ab clone did not display significant binding to AD‐2 in various assays, demonstrating the complexity of interpreting these data where antigens in different bioanalytical formats are used. Nevertheless, these data verify that the AD‐2 epitope can be targeted for the development of highly efficient mAbs for HCMV treatment and support recent studies investigating this potential. The AD‐2‐specific mAb TRL345 has produced promising preclinical results including the potential for preventing congenital transmission.^40^, ^41^ Another AD‐2 binding mAb, TCN‐202, has undergone phase 1 clinical trial and proved to be well tolerated, but further trials have not yet proceeded. The 8F9 mAb described here and previously^20^ is also a prototype biotherapeutic that will be investigated in further studies.

Our experiments also used matched human serum and saliva samples to compare the Abs for binding to gB and AD‐2 epitope. Similar to the gB vaccine study, a major observation was the much weaker IgA response to gB and AD‐2 epitope when compared to IgG responses. This occurred in both serum and saliva samples; in fact, serum results were generally indistinguishable from those found with saliva. This is somewhat surprising when considering that normal serum IgG levels are significantly higher than IgA, whereas in saliva, the opposite is true and less competition from IgG would be expected. Nevertheless, our results confirm that IgG is the dominant isotype of Abs to gB and AD‐2 in both serum and saliva from healthy individuals naturally exposed to HCMV. These data are in agreement with another study showing sensitive and specific detection of IgG to gB in saliva.^42^ Conversely, we found that human breastmilk Abs binding AD‐2 were largely of the IgA isotype, whilst a balanced IgG and IgA response to gB was observed. In fact, we observed IgA AD‐2 responses in 90% of positive human breastmilk samples as opposed to much reduced frequency of IgG responses. These data may reflect the different structural properties of IgA that exists as a monomer in serum, as does IgG, and dimeric or polymeric mucosal secretory IgA that is predominantly found in breastmilk.^43^ Thus, differences in the relative concentration and structure of IgG and IgA between serum, saliva and breastmilk may account for their variable binding affinity to AD‐2. More generally, this preponderance of IgA antibodies against HCMV in breastmilk may be an important component of passive immunity transferred between mother and child.

An additional important observation in the study was that all HCMV‐sero‐positive individuals (defined as producing IgG to gB) did not necessarily produce IgG or IgA to AD‐2. In fact, samples that contained IgG to gB displayed variable AD‐2 binding with a reduced proportion of those considered positive. These data are in agreement with previous studies that have shown approximately 50% of individuals produce antibodies to AD‐2.^44^, ^45^ It is speculated that AD‐2 binding Abs are restricted in nature due to limited structural constraints allowing antigen recognition.^19^, ^46^ This phenomenon can also help explain the reduced frequency and ability of serum and salivary IgA to bind AD‐2. In fact, we observed true IgA AD‐2 response in only one saliva sample and just four serum samples out of a total of twenty. However, human breastmilk studies indicated a dominant IgA response to AD‐2 epitope as compared to IgG in sero‐positive individuals.

We have demonstrated in breastmilk significant human IgA responses to AD‐2, an important neutralizing epitope of HCMV, and determined that IgA binding is capable of neutralizing HCMV in vitro. This may indicate a function for neonatal protection against HCMV conferred by IgA binding AD‐2. Studies using saliva and serum samples, including those obtained from a recombinant gB vaccine trial, displayed much weaker IgA binding to AD‐2, which we conclude is due to competition with the higher IgG levels present. In sero‐negative gB vaccine recipients, we could not detect IgA or IgG to AD‐2, despite the generation of both IgA and IgG to gB with this vaccination regimen, demonstrating the immunodominance of other gB epitopes. The protective immune response to HCMV is clearly very complex, requiring both cellular and humoral immunity.^47^ It is also well known that Ab responses to the AD‐2 epitope of gB are extremely variable and inconsistent. We report that IgA to AD‐2 is, numerically, a minor component of HCMV humoral immunity but is protective and warrants further investigation, in particular with respect to induction via vaccination strategies. The AD‐2 epitope can also be targeted for the development of potent therapeutic mAbs as an effective treatment option for HCMV, including those of the IgA isotype. Targeted Ab cocktails consisting of several Abs with different specificities and properties may therefore find utility in HCMV treatment.

## CONFLICT OF INTEREST

The authors declare no competing interests.

## AUTHOR CONTRIBUTIONS

SS obtained samples, performed experimental work, analysed data, wrote initial paper draft and revised the text; SH performed experimental work; HG provided samples; ACG performed experimental work; JH performed experimental work; MRM performed experimental work; MBR analysed data, provided samples, and revised and wrote paper draft; and GRM conceived experimental work, analysed data, and wrote and revised the paper.

## Supporting information


**Figure S1.** Cut off graphs of four human serum samples determined to be negative at 1:100 dilution for IgG to gB, IgA to gB, IgG to AD‐2 and IgA to AD‐2. Cut offs were calculated at each dilution based on the 2 standard deviations above the mean of all the OD values obtained from replicate assays of all 4 samples. Tables demonstrate cut off values obtained for all samples at each dilution for the same 4 samples in various assays.

**Figure S2.** Serum samples from seropositive recipients of the recombinant gB vaccination trial were analysed for IgG and IgA binding to AD‐2 by ELISA. Only individuals where one sample is considered positive (according to cut off values obtained in SFig 1) are shown. Statistical differences between the mean OD values of day 0 and day 56 of placebo and vaccine recipients for IgG and IgA for binding to AD‐2 epitope were obtained from Mann‐Whitney test (ns, not significant; *P < 0.05). Placebo groups for IgA showed no positive samples and are not included.

**Figure S3.** Cut off graphs of four undiluted human breast milk samples determined to be negative for IgG to gB, IgA to gB, IgG to AD‐2 and IgA to AD‐2. Cut off values are determined as the mean +2SD of all 4 samples. The table displays all cut off values obtained at the various dilutions of breast milk.

**Figure S4A.** Usage of kappa (k) and lambda (l) L‐chains by undiluted human milk antibodies binding to gB and AD‐2 epitope. Statistical differences between the mean OD values of antibodies containing the indicated L‐chains for binding to gB and AD‐2 epitope was obtained by Mann‐Whitney test (**** P < .0001). **Figure S4B.** Correlations of kappa (k) and lambda (l) Lchains usage by human milk antibodies binding to gB and AD‐2 epitope. Vertical (AD‐2) and horizontal(gB) dotted lines represent cut offs for negative kappa and negative lambda samples respectively. Spearman’s correlations and p values are shown.

**Figure S5A.** Cells profile SSC v FSC and gate application (black box) of unstained NS0 cells (upper) and NS0 cells expressing gB‐NT (lower). **Figure S5B.** Cell surface expression of gB‐NT. Binding of human recombinant monoclonal antibody 8F9 IgG to parental NS0 cells (upper) and NS0 cells expressing gB‐NT (lower). Black line represents unstained cells, red represents fluorescence of cells stained with secondary Ab alone (anti‐human kappa FITC) and blue represents cells stained with 8F9 IgG and secondary Ab.

## Data Availability

The authors agree to share raw data upon reasonable request.

## References

[1] Staras SA , Dollard SC , Radford KW , Flanders WD , Pass RF , Cannon MJ . Seroprevalence of cytomegalovirus infection in the United States, 1988–1994. Clin Infect Dis. 2006;43:1143–51. 17029132 10.1086/508173

[2] Liu J , Kong J , Chang YJ , Chen H , Chen YH , Han W , et al. Patients with refractory cytomegalovirus (CMV) infection following allogeneic haematopoietic stem cell transplantation are at high risk for CMV disease and non‐relapse mortality. Clin Microbiol Infect. 2015;21:e9–15. 26093077 10.1016/j.cmi.2015.06.009

[3] Kempen JH , Martin BK , Wu AW , Barron B , Thorne JE , Jabs DA . The effect of cytomegalovirus retinitis on the quality of life of patients with AIDS in the era of highly active antiretroviral therapy. Ophthalmology 2003;110:987–95. 12750102 10.1016/S0161-6420(03)00089-7

[4] Ross SA , Boppana SB . Congenital cytomegalovirus infection: outcome and diagnosis. Semin Pediatr Infect Dis. 2005;16:44–9. 15685149 10.1053/j.spid.2004.09.011

[5] van Zuylen WJ , Hamilton ST , Naing Z , Hall B , Shand A , Rawlinson WD . Congenital cytomegalovirus infection: clinical presentation, epidemiology, diagnosis and prevention. Obstet Med. 2014;7:140–6. 27512442 10.1177/1753495X14552719 PMC4934990

[6] El Helou G , Razonable RR . Safety considerations with current and emerging antiviral therapies for cytomegalovirus infection in transplantation. Exp Opin Drug Saf. 2019;18:1017–30. 10.1080/14740338.2019.1662787 31478398

[7] Ohlin M , Söderberg‐Nauclér C . Human antibody technology and the development of antibodies against cytomegalovirus. Mol Immunol. 2015;67(2 Pt A):153–70. 25802091 10.1016/j.molimm.2015.02.026

[8] Cui X , Snapper CM . Development of novel vaccines against human cytomegalovirus. Hum Vaccin Immunother. 2019;15:2673–83. 31017831 10.1080/21645515.2019.1593729 PMC6930071

[9] Revello MG , Lazzarotto T , Guerra B , Spinillo A , Ferrazzi E , Kustermann A , et al. A randomized trial of hyperimmune globulin to prevent congenital cytomegalovirus. N Engl J Med. 2014;370:1316–26. 24693891 10.1056/NEJMoa1310214

[10] Grossi P , Mohacsi P , Szabolcs Z , Potena L . Cytomegalovirus immunoglobulin after thoracic transplantation: an overview. Transplantation. 2016;100(Suppl 3):S1–4. 10.1097/TP.0000000000001094 PMC4764015 26900989

[11] Shepard HM , Phillips GL , D Thanos C , Feldmann M . Developments in therapy with monoclonal antibodies and related proteins. Clin Med. 2017;17:220–32. 10.7861/clinmedicine.17-3-220 PMC6297577 28572223

[12] Wille PT , Wisner TW , Ryckman B , Johnson DC . Human cytomegalovirus (HCMV) glycoprotein gB promotes virus entry in trans acting as the viral fusion protein rather than as a receptor‐binding protein. MBio 2013;4:e00332–13. 23736286 10.1128/mBio.00332-13 PMC3685210

[13] Vanarsdall AL , Johnson DC . Human cytomegalovirus entry into cells. Curr Opin Virol. 2012;2:37–42. 22440964 10.1016/j.coviro.2012.01.001 PMC3880194

[14] de Sousa‐Pereira P , Woof JM . IgA: structure, function, and developability. Antibodies. 2019;8:57. 10.3390/antib8040057 PMC6963396 31817406

[15] Foglierini M , Marcandalli J , Perez L . HCMV envelope glycoprotein diversity demystified. Front Microbiol. 2019;10:1005. 31156572 10.3389/fmicb.2019.01005 PMC6529531

[16] Potzsch S , Spindler N , Wiegers AK , Fisch T , Rucker P , Sticht H , et al. B cell repertoire analysis identifies new antigenic domains on glycoprotein B of human cytomegalovirus which are target of neutralizing antibodies. PLoS Pathog. 2011;7:e1002172. 21852946 10.1371/journal.ppat.1002172 PMC3154849

[17] Wagner B , Kropff B , Kalbacher H , Britt W , Sundqvist VA , Ostberg L , et al. A continuous sequence of more than 70 amino acids is essential for antibody binding to the dominant antigenic site of glycoprotein gp58 of human cytomegalovirus. J Virol. 1992;66:5290–7. 1323695 10.1128/jvi.66.9.5290-5297.1992 PMC289083

[18] Speckner A , Glykofrydes D , Ohlin M , Mach M . Antigenic domain 1 of human cytomegalovirus glycoprotein B induces a multitude of different antibodies which, when combined, results in incomplete virus neutralization. J Gen Virol. 1999;80(Pt 8):2183–91. 10466818 10.1099/0022-1317-80-8-2183

[19] Schrader JW , McLean GR . Location, location, timing: analysis of cytomegalovirus epitopes for neutralizing antibodies. Immunol Lett. 2007;112:58–60. 17714794 10.1016/j.imlet.2007.07.001

[20] McLean GR , Olsen OA , Watt IN , Rathanaswami P , Leslie KB , Babcook JS , et al. Recognition of human cytomegalovirus by human primary immunoglobulins identifies an innate foundation to an adaptive immune response. J Immunol. 1950;2005:4768–78. 10.4049/jimmunol.174.8.4768 15814702

[21] Ohlin M . A new look at a poorly immunogenic neutralization epitope on cytomegalovirus glycoprotein B. Is there cause for antigen redesign? Mol Immunol. 2014;60:95–102. 24802891 10.1016/j.molimm.2014.03.015

[22] Smith K , Shah H , Muther JJ , Duke AL , Haley K , James JA . Antigen nature and complexity influence human antibody light chain usage and specificity. Vaccine. 2016;34:2813–20. 27113164 10.1016/j.vaccine.2016.04.040 PMC4876604

[23] Manghera A , McLean GR . Human cytomegalovirus vaccination: progress and perspectives of recombinant gB. Future Virol. 2016;11:439–49.

[24] Frey SE , Harrison C , Pass RF , Yang E , Boken D , Sekulovich RE , et al. Effects of antigen dose and immunization regimens on antibody responses to a cytomegalovirus glycoprotein B subunit vaccine. J Infect Dis. 1999;180:1700–3. 10515836 10.1086/315060

[25] Mitchell DK , Holmes SJ , Burke RL , Duliege AM , Adler SP . Immunogenicity of a recombinant human cytomegalovirus gB vaccine in seronegative toddlers. Pediatr Infect Dis J. 2002;21:133–8. 11840080 10.1097/00006454-200202000-00009

[26] Sabbaj S , Pass RF , Goepfert PA , Pichon S . Glycoprotein B vaccine is capable of boosting both antibody and CD4 T‐cell responses to cytomegalovirus in chronically infected women. J Infect Dis. 2011;203:1534–41. 21592981 10.1093/infdis/jir138 PMC3096785

[27] Pass RF , Duliege AM , Boppana S , Sekulovich R , Percell S , Britt W , et al. A subunit cytomegalovirus vaccine based on recombinant envelope glycoprotein B and a new adjuvant. J Infect Dis. 1999;180:970–5. 10479120 10.1086/315022

[28] Bernstein DI , Munoz FM , Callahan ST , Rupp R , Wootton SH , Edwards KM , et al. Safety and efficacy of a cytomegalovirus glycoprotein B (gB) vaccine in adolescent girls: a randomized clinical trial. Vaccine 2016;34:313–9. 26657184 10.1016/j.vaccine.2015.11.056 PMC4701617

[29] Pass RF , Zhang C , Evans A , Simpson T , Andrews W , Huang ML , et al. Vaccine prevention of maternal cytomegalovirus infection. N Engl J Med. 2009; 360:1191–9. 19297572 10.1056/NEJMoa0804749 PMC2753425

[30] Griffiths PD , Stanton A , McCarrell E , Smith C , Osman M , Harber M , et al. Cytomegalovirus glycoprotein‐B vaccine with MF59 adjuvant in transplant recipients: a phase 2 randomised placebo‐controlled trial. Lancet 2011;377:1256–63. 21481708 10.1016/S0140-6736(11)60136-0 PMC3075549

[31] Baraniak I , Kropff B , Ambrose L , McIntosh M , McLean GR , Pichon S , et al. Protection from cytomegalovirus viremia following glycoprotein B vaccination is not dependent on neutralizing antibodies. Proc Natl Acad Sci USA. 2018;115:6273–8. 29686064 10.1073/pnas.1800224115 PMC6004462

[32] Nelson CS , Huffman T , Jenks JA , Cisneros de la Rosa E , Xie G , Vandergrift N , et al. HCMV glycoprotein B subunit vaccine efficacy mediated by nonneutralizing antibody effector functions. Proc Natl Acad Sci USA. 2018;115:6267–72. 29712861 10.1073/pnas.1800177115 PMC6004431

[33] Baraniak I , Kropff B , McLean GR , Pichon S , Piras‐Douce F , Milne RSB , et al. Epitope‐specific humoral responses to human cytomegalovirus glycoprotein‐B vaccine with MF59: anti‐AD2 levels correlate with protection from viremia. J Infect Dis. 2018; 217:1907–17. 29528415 10.1093/infdis/jiy102 PMC5972559

[34] Bialas KM , Westreich D , Cisneros de la Rosa E , Nelson CS , Kauvar LM , Fu TM , et al. Maternal antibody responses and nonprimary congenital cytomegalovirus infection of HIV‐1‐exposed infants. J Infect Dis. 2016;214:1916–23. 27923951 10.1093/infdis/jiw487 PMC5142097

[35] Baraniak I , Kern F , Holenya P , Griffiths P , Reeves M . Original antigenic sin shapes the immunological repertoire evoked by human cytomegalovirus glycoprotein B/MF59 vaccine in seropositive recipients. J Infect Dis. 2019; 220:228–32. 30815685 10.1093/infdis/jiz089 PMC6581893

[36] McLean GR , Nakouzi A , Casadevall A , Green NS . Human and murine immunoglobulin expression vector cassettes. Mol Immunol. 2000;37:837–45. 11257305 10.1016/s0161-5890(00)00101-2

[37] Plomp R , de Haan N , Bondt A , Murli J , Dotz V , Wuhrer M . Comparative glycomics of immunoglobulin A and G From saliva and plasma reveals biomarker potential. Front Immunol. 2018;9:2436. 30405629 10.3389/fimmu.2018.02436 PMC6206042

[38] McCutcheon KM , Gray J , Chen NY , Liu K , Park M , Ellsworth S , et al. Multiplexed screening of natural humoral immunity identifies antibodies at fine specificity for complex and dynamic viral targets. mAbs. 2014;6:460–73. 24492306 10.4161/mabs.27760 PMC3984334

[39] McLean GR , Torres M , Elguezabal N , Nakouzi A , Casadevall A . Isotype can affect the fine specificity of an antibody for a polysaccharide antigen. J Immunol. 1950;2002:1379–86. 10.4049/jimmunol.169.3.1379 12133962

[40] Kauvar LM , Liu K , Park M , DeChene N , Stephenson R , Tenorio E , et al. A high‐affinity native human antibody neutralizes human cytomegalovirus infection of diverse cell types. Antimicrob Agents Chemother. 2015;59:1558–68. 25534746 10.1128/AAC.04295-14 PMC4325823

[41] McVoy MM , Tenorio E , Kauvar LM . A native human monoclonal antibody targeting HCMV gB (AD‐2 Site I). Int J Mol Sci. 2018;19:3982. 10.3390/ijms19123982 PMC6321246 30544903

[42] Wang JB , Adler SP . Salivary antibodies to cytomegalovirus (CMV) glycoprotein B accurately predict CMV infections among preschool children. J Clin Microbiol. 1996;34:2632–4. 8880541 10.1128/jcm.34.10.2632-2634.1996 PMC229342

[43] Van de Perre P . Transfer of antibody via mother's milk. Vaccine. 2003;21:3374–6. 12850343 10.1016/s0264-410x(03)00336-0

[44] Meyer H , Sundqvist VA , Pereira L , Mach M . Glycoprotein gp116 of human cytomegalovirus contains epitopes for strain‐common and strain‐specific antibodies. J Gen Virol. 1992;73(Pt 9):2375–83. 1383409 10.1099/0022-1317-73-9-2375

[45] Kropff B , Landini MP , Mach M . An ELISA using recombinant proteins for the detection of neutralizing antibodies against human cytomegalovirus. J Med Virol. 1993;39:187–95. 8385703 10.1002/jmv.1890390303

[46] Thomson CA , Bryson S , McLean GR , Creagh AL , Pai EF , Schrader JW . Germline V‐genes sculpt the binding site of a family of antibodies neutralizing human cytomegalovirus. EMBO J. 2008;27:2592–602. 18772881 10.1038/emboj.2008.179 PMC2567409

[47] Jackson SE , Mason GM , Wills MR . Human cytomegalovirus immunity and immune evasion. Virus Res. 2011;157:151–60. 21056604 10.1016/j.virusres.2010.10.031

