# Supplementary material for: IgA binds to the AD‐2 epitope of glycoprotein B and neutralizes human cytomegalovirus
Source: Immunology. 2020 Dec 13;162(3):314–27. doi: 10.1111/imm.13286 (PMC7884650; doi:10.1111/imm.13286)
Supplement: Supplementary file 1 — Figure S1. Cut off graphs of four human serum samples determined to be negative at 1:100 dilution for IgG to gB, IgA to gB, IgG to AD‐2 and IgA to AD‐2. Cut offs were calculated at each dilution based on the 2 standard deviations above the mean of all the OD values obtained from replicate assays of all 4 samples. Tables demonstrate cut off values obtained for all samples at each dilution for the same 4 samples in various assays. [file IMM-162-314-s001.pptx]

## Slide 1
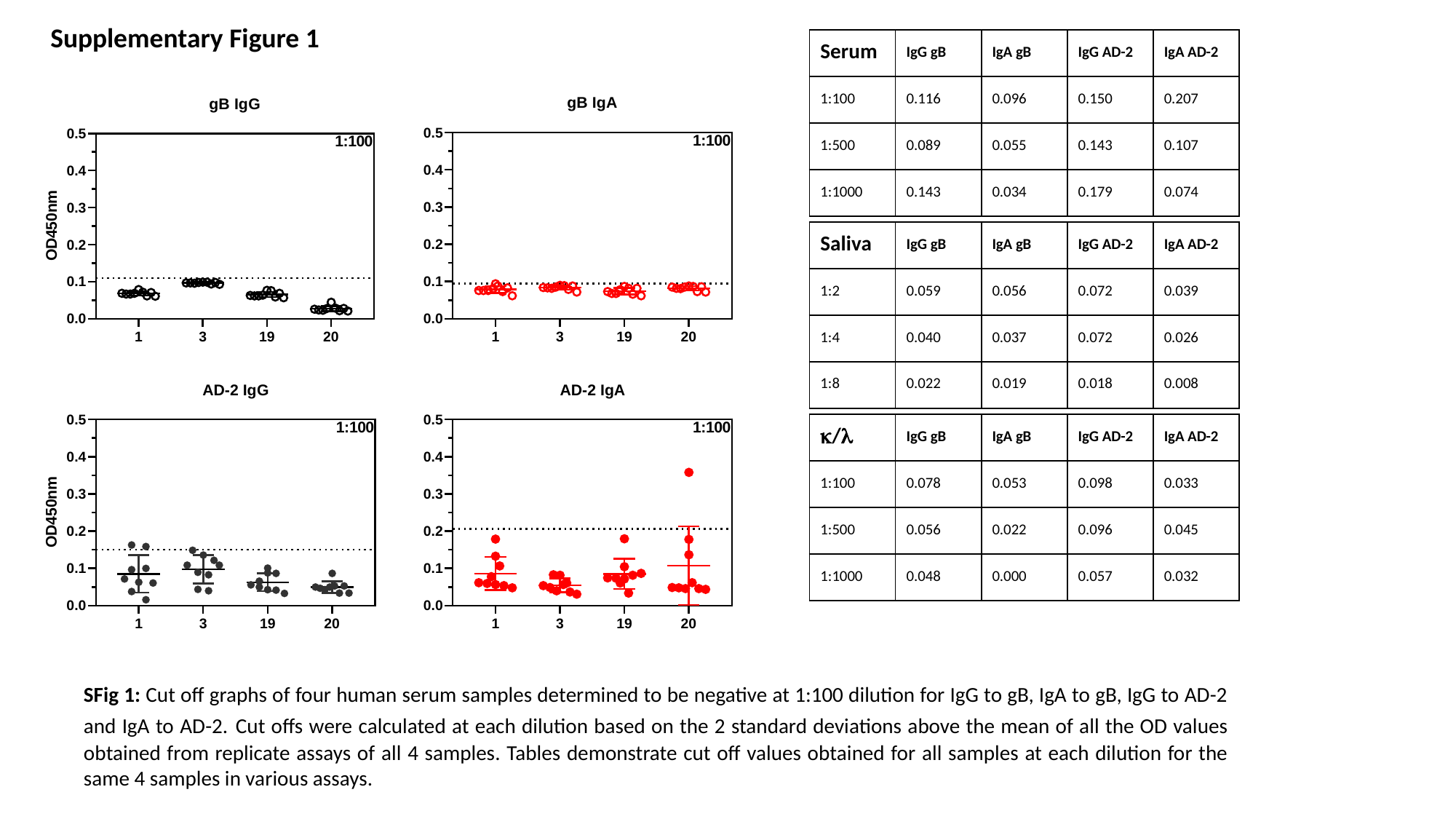

Supplementary Figure 1
| Serum | IgG gB | IgA gB | IgG AD-2 | IgA AD-2 |
| --- | --- | --- | --- | --- |
| 1:100 | 0.116 | 0.096 | 0.150 | 0.207 |
| 1:500 | 0.089 | 0.055 | 0.143 | 0.107 |
| 1:1000 | 0.143 | 0.034 | 0.179 | 0.074 |
| Saliva | IgG gB | IgA gB | IgG AD-2 | IgA AD-2 |
| --- | --- | --- | --- | --- |
| 1:2 | 0.059 | 0.056 | 0.072 | 0.039 |
| 1:4 | 0.040 | 0.037 | 0.072 | 0.026 |
| 1:8 | 0.022 | 0.019 | 0.018 | 0.008 |
| k/l | IgG gB | IgA gB | IgG AD-2 | IgA AD-2 |
| --- | --- | --- | --- | --- |
| 1:100 | 0.078 | 0.053 | 0.098 | 0.033 |
| 1:500 | 0.056 | 0.022 | 0.096 | 0.045 |
| 1:1000 | 0.048 | 0.000 | 0.057 | 0.032 |
SFig 1: Cut off graphs of four human serum samples determined to be negative at 1:100 dilution for IgG to gB, IgA to gB, IgG to AD-2 and IgA to AD-2. Cut offs were calculated at each dilution based on the 2 standard deviations above the mean of all the OD values obtained from replicate assays of all 4 samples. Tables demonstrate cut off values obtained for all samples at each dilution for the same 4 samples in various assays.
