# Supplementary material for: IgA binds to the AD‐2 epitope of glycoprotein B and neutralizes human cytomegalovirus
Source: Immunology. 2020 Dec 13;162(3):314–27. doi: 10.1111/imm.13286 (PMC7884650; doi:10.1111/imm.13286)
Supplement: Supplementary file 2 — Figure S2. Serum samples from seropositive recipients of the recombinant gB vaccination trial were analysed for IgG and IgA binding to AD‐2 by ELISA. Only individuals where one sample is considered positive (according to cut off values obtained in SFig 1) are shown. Statistical differences between the mean OD values of day 0 and day 56 of placebo and vaccine recipients for IgG and IgA for binding to AD‐2 epitope were obtained from Mann‐Whitney test (ns, not significant; *P < 0.05). Placebo groups for IgA showed no positive samples and are not included. [file IMM-162-314-s002.pptx]

## Slide 1
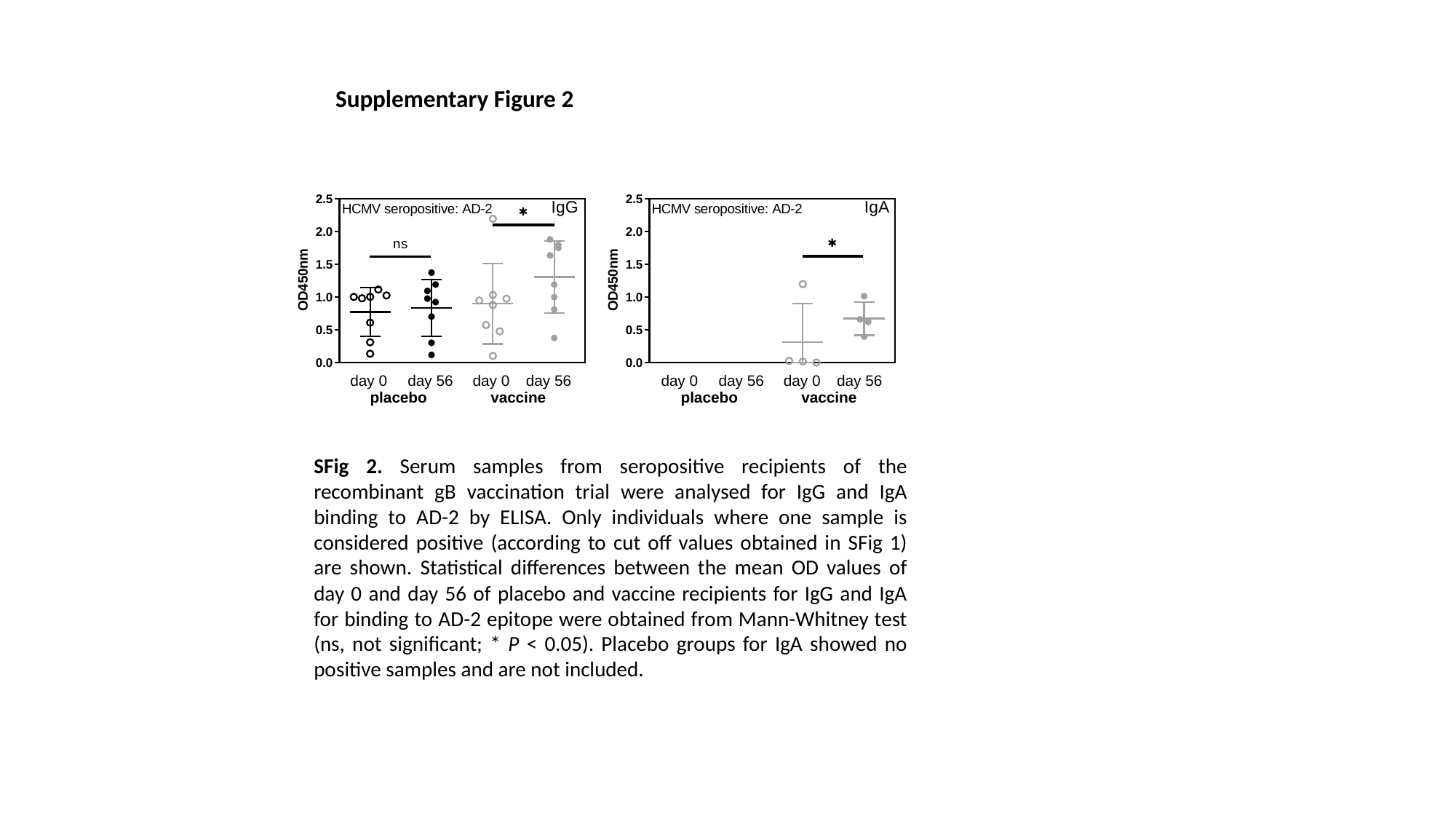

Supplementary Figure 2
IgA
IgG
day 0
day 56
day 0
day 56
placebo
vaccine
day 0
day 56
day 0
day 56
placebo
vaccine
SFig 2. Serum samples from seropositive recipients of the recombinant gB vaccination trial were analysed for IgG and IgA binding to AD-2 by ELISA. Only individuals where one sample is considered positive (according to cut off values obtained in SFig 1) are shown. Statistical differences between the mean OD values of day 0 and day 56 of placebo and vaccine recipients for IgG and IgA for binding to AD-2 epitope were obtained from Mann-Whitney test (ns, not significant; * P < 0.05). Placebo groups for IgA showed no positive samples and are not included.
