# Supplementary material for: IgA binds to the AD‐2 epitope of glycoprotein B and neutralizes human cytomegalovirus
Source: Immunology. 2020 Dec 13;162(3):314–27. doi: 10.1111/imm.13286 (PMC7884650; doi:10.1111/imm.13286)
Supplement: Supplementary file 3 — Figure S3. Cut off graphs of four undiluted human breast milk samples determined to be negative for IgG to gB, IgA to gB, IgG to AD‐2 and IgA to AD‐2. Cut off values are determined as the mean +2SD of all 4 samples. The table displays all cut off values obtained at the various dilutions of breast milk. [file IMM-162-314-s003.pptx]

## Slide 1
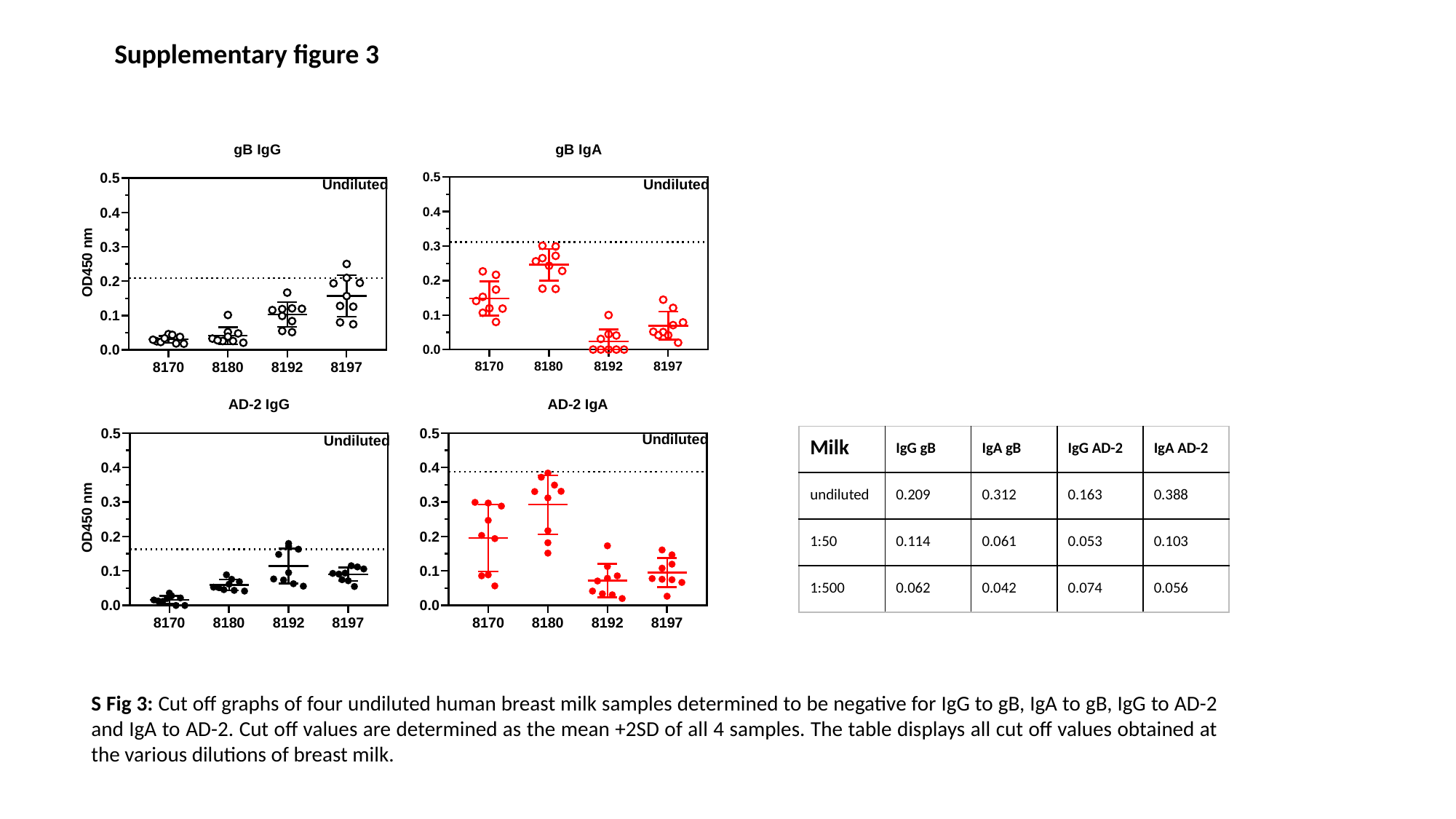

Supplementary figure 3
| Milk | IgG gB | IgA gB | IgG AD-2 | IgA AD-2 |
| --- | --- | --- | --- | --- |
| undiluted | 0.209 | 0.312 | 0.163 | 0.388 |
| 1:50 | 0.114 | 0.061 | 0.053 | 0.103 |
| 1:500 | 0.062 | 0.042 | 0.074 | 0.056 |
S Fig 3: Cut off graphs of four undiluted human breast milk samples determined to be negative for IgG to gB, IgA to gB, IgG to AD-2 and IgA to AD-2. Cut off values are determined as the mean +2SD of all 4 samples. The table displays all cut off values obtained at the various dilutions of breast milk.
