# Supplementary material for: IgA binds to the AD‐2 epitope of glycoprotein B and neutralizes human cytomegalovirus
Source: Immunology. 2020 Dec 13;162(3):314–27. doi: 10.1111/imm.13286 (PMC7884650; doi:10.1111/imm.13286)
Supplement: Supplementary file 4 — Figure S4A. Usage of kappa (k) and lambda (l) L‐chains by undiluted human milk antibodies binding to gB and AD‐2 epitope. Statistical differences between the mean OD values of antibodies containing the indicated L‐chains for binding to gB and AD‐2 epitope was obtained by Mann‐Whitney test (**** P < .0001). Figure S4B. Correlations of kappa (k) and lambda (l) Lchains usage by human milk antibodies binding to gB and AD‐2 epitope. Vertical (AD‐2) and horizontal(gB) dotted lines represent cut offs for negative kappa and negative lambda samples respectively. Spearman’s correlations and p values are shown. [file IMM-162-314-s004.pdf]

Supplementary figure 4

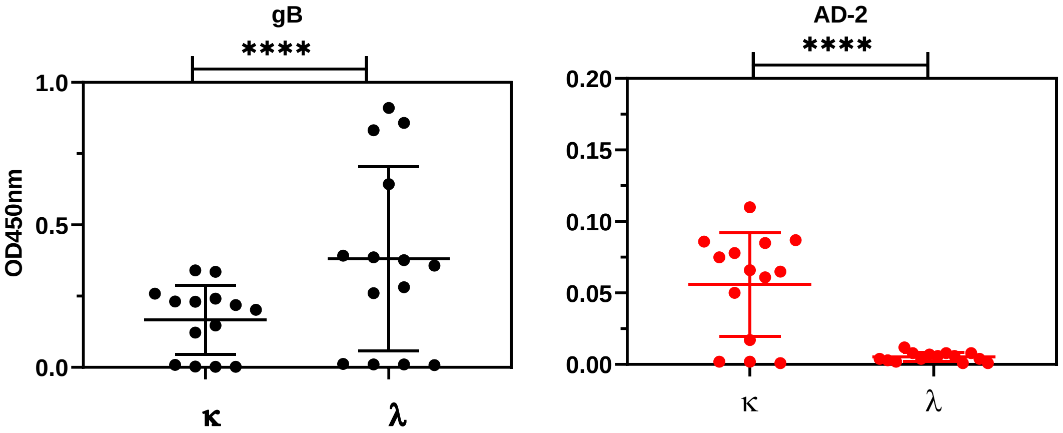

S Fig 4A: Usage of kappa (κ) and lambda (λ) L-chains by undiluted human milk antibodies binding to gB and AD-2 epitope. Statistical differences between the mean OD values of antibodies containing the indicated L-chains for binding to gB and AD-2 epitope was obtained by Mann-Whitney test (***** P* < .0001).

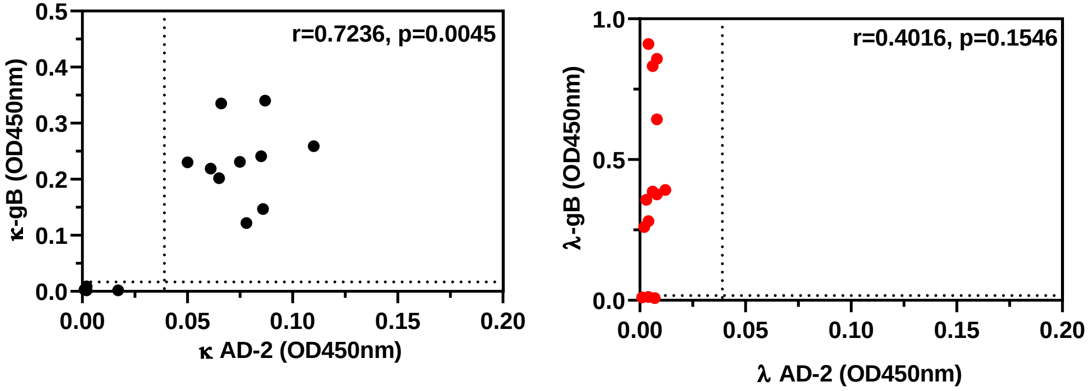

S Fig 4B: Correlations of kappa (κ) and lambda (λ) L-chains usage by human milk antibodies binding to gB and AD-2 epitope. Vertical (AD-2) and horizontal (gB) dotted lines represent cut offs for negative kappa and negative lambda samples respectively. Spearman's correlations and p values are shown.
