# Supplementary material for: IgA binds to the AD‐2 epitope of glycoprotein B and neutralizes human cytomegalovirus
Source: Immunology. 2020 Dec 13;162(3):314–27. doi: 10.1111/imm.13286 (PMC7884650; doi:10.1111/imm.13286)
Supplement: Supplementary file 5 — Figure S5A. Cells profile SSC v FSC and gate application (black box) of unstained NS0 cells (upper) and NS0 cells expressing gB‐NT (lower). Figure S5B. Cell surface expression of gB‐NT. Binding of human recombinant monoclonal antibody 8F9 IgG to parental NS0 cells (upper) and NS0 cells expressing gB‐NT (lower). Black line represents unstained cells, red represents fluorescence of cells stained with secondary Ab alone (anti‐human kappa FITC) and blue represents cells stained with 8F9 IgG and secondary Ab. [file IMM-162-314-s005.pptx]

## Slide 1
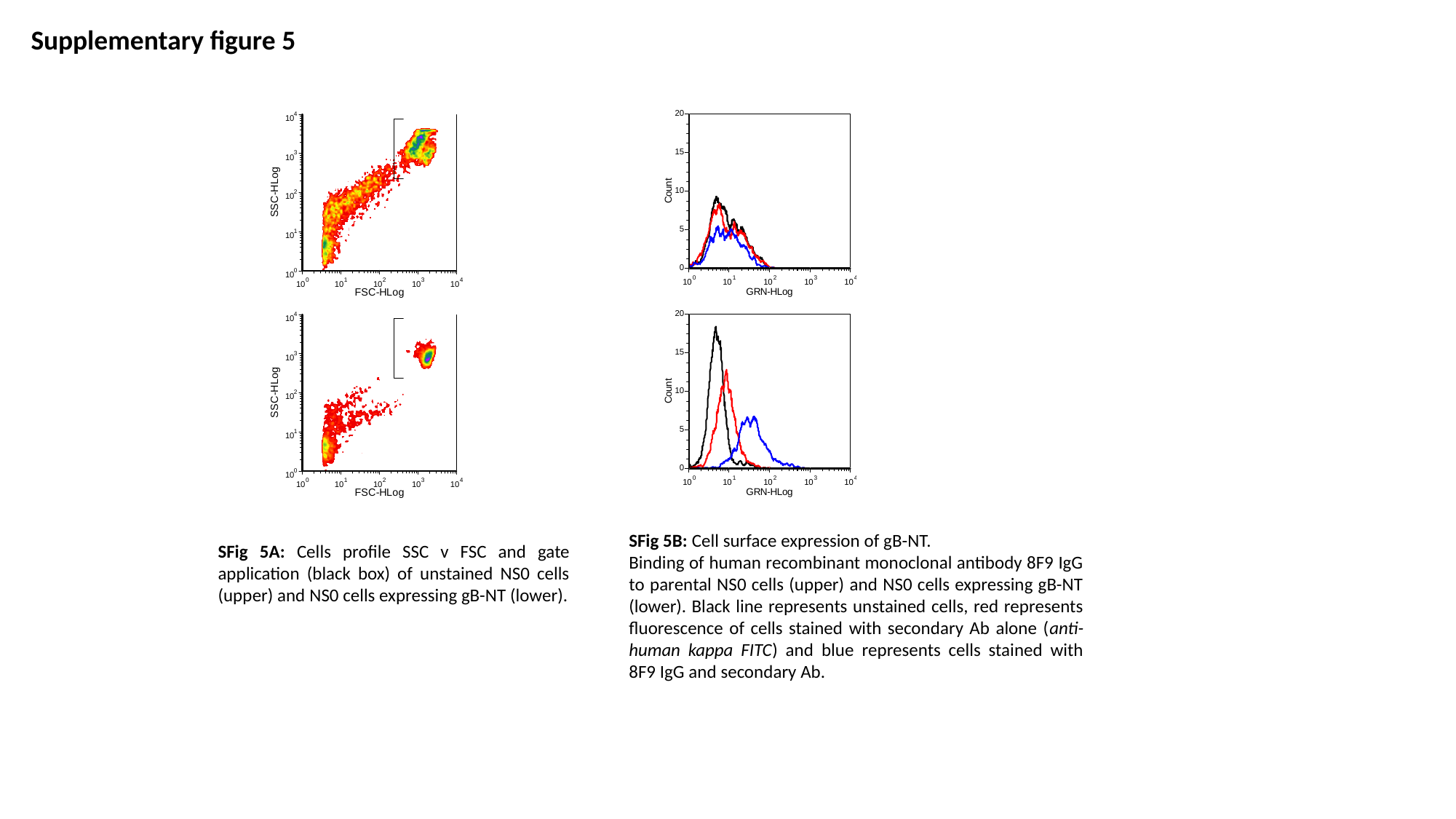

Supplementary figure 5
SFig 5B: Cell surface expression of gB-NT.
Binding of human recombinant monoclonal antibody 8F9 IgG to parental NS0 cells (upper) and NS0 cells expressing gB-NT (lower). Black line represents unstained cells, red represents fluorescence of cells stained with secondary Ab alone (anti-human kappa FITC) and blue represents cells stained with 8F9 IgG and secondary Ab.
SFig 5A: Cells profile SSC v FSC and gate application (black box) of unstained NS0 cells (upper) and NS0 cells expressing gB-NT (lower).
